# Supplementary material for: Determination of the Prevalence of Microsatellite Instability, BRAF and KRAS/NRAS Mutation Status in Patients with Colorectal Cancer in Slovakia
Source: Cancers (Basel). 2024 Mar 12;16(6):1128. doi: 10.3390/cancers16061128 (PMC10969032; doi:10.3390/cancers16061128)
Supplement: Supplementary file 1 [file cancers-16-01128-s001.zip › cancers-2866278-supplementary.pdf]

Supplementary Table S1: List of all patients enrolled in the study with their respective parameters: Patient number in the study, code of diagnosis (according to ICD-10), age, gender, neoplastic cells percentage (NCP) in a tissue sample, mutation status of *RAS*, *BRAF*, and MSI status.

Patient	ICD-10 code	Age	Sex	NCP (%):	KRAS/NRAS	BRAF	MSI
1	C20	86	M	20	KRAS: c.38G>T, p.G13V	N	MSS
2	C20	79	F	70	KRAS: c.436G>C, p.A146P	N	MSS
3	C18.7	62	F	80	KRAS: c.38G>A, p.G13D	N	MSS
4	C20	71	M	70	N	N	MSS
5	C19	56	F	60	N	N	MSS
6	C18.2	62	F	100	KRAS: c.38G>A, p.G13D	N	MSI-H
7	C20	78	M	20	NRAS: c.183A>T, p.Q61H	N	MSS
8	C18.7	65	F	30	N	N	MSS
9	C18.5	45	F	40	N	N	MSS
10	C18.0	44	M	20	KRAS: c.34G>A, p.G12S	N	MSS
11	C18.9	65	M	80	N	N	MSS
12	C20	55	M	10	N	N	MSS
13	C18.5	70	M	20	N	N	MSS
14	C19	61	M	60	N	p.V600E	MSS
15	C19	51	M	80	N	N	MSS
16	C20	42	F	70	N	N	MSS
17	C19	56	F	10	N	N	MSS
18	C20	27	M	70	N	N	MSS
19	C20	72	F	40	N	N	MSS
20	C18.9	69	F	70	N	N	MSS
21	C19	66	M	90	N	N	MSS
22	C20	77	F	20	N	N	MSS
23	C20	73	M	60	N	N	MSS
24	C21.0	68	F	70	c.183A>C, p.Q61H	N	MSS
25	C20	70	M	60	N	N	MSS
26	C20	58	F	80	KRAS: c.35G>T, p.G12V	N	MSS
27	C19	70	M	70	N	p.V600E	MSS
28	C20	75	M	20	N	N	MSS
29	C26.0	65	M	70	N	N	MSS
30	C20	47	F	90	KRAS: c.35G>T, p.G12V	N	MSS
31	C18	74	M	80	N	p.V600E	MSI-H
32	C19	57	M	90	KRAS: c.35G>T, p.G12V	N	MSS
33	C18.7	51	M	70	N	N	MSS
34	C19	57	M	70	N	N	MSS
35	C18.2	79	F	70	KRAS: c.35G>A, p.G12D	p.V600E	MSI-H
36	C18.7	65	M	70	KRAS: c.35G>T, p.G12V	N	MSS
37	C18.2	67	M	90	KRAS: c.35G>A, p.Gly12Asp	N	MSI-H
38	C18.7	70	F	70	NRAS: c.183A>T, p.Gln61His	N	MSS
39	C18.7	44	M	30	N	N	MSS
40	C20	64	M	40	KRAS: c.35G>A, p.Gly12Asp	N	MSS
41	C21.0	70	M	20	N	N	MSS

42	C20	80	F	60	N	N	MSS
43	C19	67	F	70	KRAS: c.35G>T, p.Gly12Val	N	MSS
44	C20	67	M	20	N	N	MSS
45	C18	61	M	70	KRAS: c.35G>T, p.Gly12Val	N	MSS
46	C20	66	M	20	KRAS: c.34G>T, p.Gly12Cys	N	MSS
47	C20	26	M	20	N	N	MSS
48	C18.0	65	F	40	KRAS: c.35G>A, p.Gly12Asp	N	MSS
49	C19	59	M	70	N	N	MSS
50	C18.7	67	F	30	N	N	MSS
51	C18.7	62	F	50	N	N	MSS
52	C20	62	F	30	NRAS: c.35G>C, p.Gly12Ala	N	MSS
53	C18.8	70	F	50	N	N	MSS
54	C18.0	70	F	5	KRAS: c.35G>T, p.Gly12Val	N	MSS
55	C21.0	73	M	30	KRAS: c.35G>T, p.Gly12Val	N	MSS
56	C18.0	60	F	70	KRAS: c.38G>A, p.Gly13Asp	N	MSS
57	C18.9	74	M	70	N	N	MSS
58	C18.2	77	F	60	N	p.V600E	MSS
59	C18.7	64	M	20	N	N	MSS
60	C20	64	M	20	N	N	MSS
61	C18.9	57	M	5	N	N	MSS
62	C20	72	F	15	KRAS: c.35G>A, p.G12D	N	MSS
63	C20	66	F	10	N	N	MSS
64	C20	43	M	70	NRAS: c.35G>A, p.G12D	N	MSS
65	C18	39	F	8	KRAS: c.35G>A, p.G12D	N	MSS
66	C20	61	F	70	NRAS: c.182A>G, p.Q61R	N	MSS
67	C18.5	49	F	70	KRAS: c.182A>T, p.Q61L	N	MSS
68	C18.0	69	M	20	KRAS: c.35G>A, p.G12D	N	MSS
69	C18.7	77	M	10	N	N	MSS
70	C20	68	M	70	N	N	MSS
71	C19	64	M	10	N	N	MSS
72	C18.0	67	F	90	KRAS: c.38G>A, p.G13D	N	MSS
73	C18.7	51	M	90	KRAS: c.38G>A, p.G13D	N	MSS
74	C21.0	54	F	90	KRAS: c.34G>A, p.G12S	N	MSS
75	C20	78	M	20	KRAS: c.437C>T, p.A146V	N	MSS
76	C18.7	75	F	15	NRAS: c.35G>A, p.G12D	N	MSS
77	C20	59	M	85	KRAS: c.38G>A, p.G13D	N	MSS
78	C20	58	F	30	KRAS: c.35G>A, p.G12D	N	MSS
79	C21.0	42	M	80	N	N	MSS
80	C21.0	54	F	70	KRAS: c.35G>A, p.G12D	N	MSS
81	C18.7	68	M	60	KRAS: c.35G>A, p.G12D	N	MSS
82	C26.9	58	F	40	KRAS: c.35G>A, p.G12D	N	MSS
83	C26.0	42	M	80	N	N	MSS
